# A Rapid Method of the Rock Mass Surface Reconstruction for Surface Deformation Detection at Close Range

**DOI:** 10.3390/s20185371

**Published:** 2020-09-19

**Authors:** Qijun Hu, Chunlin Ma, Yu Bai, Leping He, Jie Tan, Qijie Cai, Junsen Zeng

**Affiliations:** 1 School of Civil Engineering and Geomatics, Southwest Petroleum University, Chengdu 610500, China; 201231010027@swpu.edu.cn (Q.H.); 201921000850@stu.swpu.edu.cn (C.M.); 201922000643@stu.swpu.edu.cn (J.T.); 2 School of Mechatronic Engineering, Southwest Petroleum University, Chengdu 610500, China; 201722000533@stu.swpu.edu.cn (Y.B.); 201911000062@stu.swpu.edu.cn (J.Z.); 3 School of Transportation and Logistics, Southwest Jiaotong University, Chengdu 610031, China; caiqijieswjt@my.swjtu.edu.cn

**Keywords:** rock mass, 3D reconstruction, surface deformation, improved SfM

## Abstract

Characterizing the surface deformation during the inter-survey period could assist in understanding rock mass progressive failure processes. Moreover, 3D reconstruction of rock mass surface is a crucial step in surface deformation detection. This study presents a method to reconstruct the rock mass surface at close range in a fast way using the improved structure from motion—multi view stereo (SfM) algorithm for surface deformation detection. To adapt the unique feature of rock mass surface, the AKAZE algorithm with the best performance in rock mass feature detection is introduced to improve SfM. The surface reconstructing procedure mainly consists of image acquisition, feature point detection, sparse reconstruction, and dense reconstruction. Hereafter, the proposed method was verified by three experiments. Experiment 1 showed that this method effectively reconstructed the rock mass model. Experiment 2 proved the advanced accuracy of the improved SfM compared with the traditional one in reconstructing the rock mass surface. Eventually, in Experiment 3, the surface deformation of rock mass was quantified through reconstructing images before and after the disturbance. All results have shown that the proposed method could provide reliable information in rock mass surface reconstruction and deformation detection.

## 1. Introduction

Limited to the topographic and environmental conditions, projects in Southwest China that have been or are under construction are closely related to rock masses, for example, tunnel engineering, slope engineering, and foundation engineering. Much work has been done to evaluate the properties of rock engineering by analyzing the mechanics and failure characteristics of rock masses [1,2]. The surface deformation analysis of the rock masses could also provide useful information to understand the failure mechanism and stability [3,4]. Rock mass surface deformation detection is of great significance in the safety management of a construction project, which could even predict an initial danger of rock engineering to some extent. The surface reconstruction is the basis to quantify the surface deformation process of rock engineering. This study is going to explore a rapid method of the rock mass surface three-dimensional (3D) reconstruction for surface deformation detection in a close range.

Biological monitoring is an essential means of surface deformation detection in rock engineering. In the past decade, remote surveying technology has made significant progress in rapidly acquiring 3D high-resolution digital images [2]. It is commonplace to characterize rock mass surfaces through digital photogrammetry [5,6], light detection and ranging (LiDAR) [7], Interferometric Synthetic Aperture Radar (INSAR) interferometry [8], 3D laser scanning [9,10], and unmanned aerial vehicles (UAVs) [10,11]. These techniques are suitable to measure variety of processes according to their own features. Recent developments in 3D photogrammetry could create 3D surface models or triangulated irregular networks (TINs) using multiple photos [12,13]. Owing to the economy, convenience, and intelligence, the structure from motion–multi view stereo (SfM-MVS) has attracted continuous investigations into acquiring digital information within 3D reconstruction [14,15]. The SfM calculates the object’s position based on the reference point deduced from photos, which is mainly used for sparse reconstruction. Moreover, MVS generates a broad range of point clouds based on the predicted object position and reference point, which is used for dense reconstruction. This technology has been extended to land surface changes [16,17], river erosion [18], and rock failure [19], among others. Some works even achieve a highly accurate reconstruction model that indicates SfM-MVS could provide a survey precision comparable to the current measurement methods [20,21,22,23,24].

However, the literature lacks the investigation into the surface reconstruction of rock mass at close range using SfM-MVS. The quality of 3D reconstruction is one of the most critical issues bothering rock mass surface deformation detection. First of all, the selection of verification methods would lead to different assessment results [25,26,27]. Secondly, the size of the pixel increases as the ranging increases [28], thus the reconstruction accuracy would reduce linearly with the increase in ranging. As to the error measurement criterion, root mean squared error (RMSE) is the most common method to evaluate the difference between model measurements and independent observations [25,29,30]. Last, but not least, regarding the data acquisition facility, sensors that capture different types of images may complex the results of the 3D reconstruction. To adapt the unique feature of rock mass surface, serial factors that affect the quality should be considered in the practical 3D reconstruction of rock mass surface at close range.

Characterizing the surface deformation during the inter-survey period could assist in understanding the rock mass progressive failure processes. The quality of the surface deformation detection is subject to the quality of surface reconstruction using the SfM algorithm. This study proposed an improved A-SfM 3D reconstruction algorithm to realize a more accurate rock mass surface reconstruction by obtaining a higher accuracy 3D reconstruction point cloud of rock surface. The surface deformation of rock mass was detected by 3D reconstruction at different times using an open source software, CloudCompare. This study aims at providing more accurate monitoring information in predicting the further disaster of rock engineering and helps to understand the gradual failure process of rock mass surface to some extent.

## 2. Proposed Method

The SfM, an algorithm of 3D reconstruction based on various unordered images, is introduced to reconstruct the surface of rock mass with multiple images acquired at close range. The accuracy and cost of 3D reconstruction depend on the number of feature points extracted and matched, calculation time, central processing unit (CPU) utility, and mismatched accuracy of feature points. Regarding the feature extraction part of SfM, the SIFT algorithm with scale and rotation invariance is the mainstream. However, the edge information is still probably lost because the Gaussian blur would smooth all scales of the target image to the same degree of detail and noise. To build a 3D reconstruction model that is more suitable for rock mass image characteristics, this study emphasized improving the feature extraction of the SfM algorithm.

The image data sets of rock mass were taken in Aba Tibetan, Sichuan, China. A total of 41 images were classified into four groups according to the transform of intensity, rotation, scale, and fuzzy, respectively. Table 1 and Table 2 list the parameters of five feature extraction algorithms (AKAZE, ORB, SURF, SIFT, and BRISK), including the number of feature points, number of interior points, number of matching points, execution time, CPU utility, efficiency of feature points, and matching accuracy.

Test results were analyzed comprehensively with the Entropy Weight—TOPSIS. The comprehensive evaluation index $C_{i}^{*}$ is calculated as follows:

$$C_{i}{}^{*}=\left[0.1234,\;0.1325,\;0.3304,\;0.3656,\;0.0481\right]$$

where SIFT is 0.1234, SURF is 0.1325, ORB is 0.3304, AKAZE is 0.3656, and BRISK is 0.0481. According to the comprehensive evaluation index, the five algorithms are ranked as follows: AWAKE, ORB, SURF, SIFT, and BRISK. Therefore, AKAZE was used to improve the traditional SfM-MVS.

**Figure 1:**
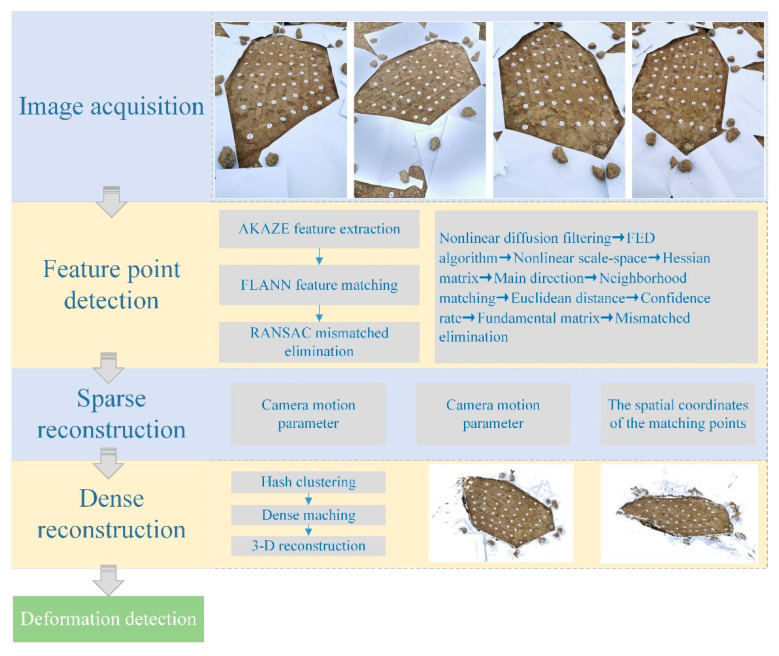
The framework for 3D reconstruction of rock mass surface. FED, fast explicit diffusion.

The framework for 3D reconstruction of rock mass surface mainly includes image acquisition, feature point detection, sparse reconstruction, and dense reconstruction, as shown in Figure 1.

## 3. Basic Theory and Methods

### 3.1. Image Acquisition

Reconstruction using SfM has less requirements for image acquisition sensors. Table 3 shows the related technical specifications of sensors for image acquisition. The primary purpose of image acquisition sensor design is to select the most appropriate sensor to acquire the image with sufficient resolution. A high-performance single-lens reflex camera is used for image acquisition, in order to mostly adapt to the actual situation of the rock engineering site and reduce time and efficient costs. Most of these cameras enable acquiring ground resolution in centimeters and have strong robustness.

### 3.2. Feature Point Detection

**Feature extraction.** The AKAZE algorithm is used to improve the SfM in surface reconstruction owing to the optimization in rock mass image characteristics. AKAZE is a feature extraction algorithm with good robustness due to the introduced modified-local difference binary (M-LDB). The main principle is as follows.

A nonlinear diffusion filter describes the variation of image brightness L in different scales using the divergence of flow function, Formula (1):

(1) ∂L/∂t=div(c(x,y,t)·∇L)

where L is the brightness matrix of the image; div and ∇ represent the divergence and the gradient of the image, respectively; c(x,y,t) is the conduction function; and t is evolutionary time.

The conduction function allows the diffusion equation to adapt to the local structure characteristics of the image. The conduction function is defined as Formula (2).

(2) $$c\left(x,y,t\right)=g\left(\left|\nabla L_{\sigma }\left(x,y,t\right)\right|\right)$$

where $\nabla L_{\sigma }$ is the image smoothed by Gaussian. The conduction kernel function is selected as Formula (3) for optimal diffusion smoothing.

(3) $$g_{2}=\frac{1}{\left(1+|\nabla L\sigma {|}^{2}/\lambda^{2}\right)}$$

where λ is the deciding factor, which is used to control the degree of diffusion and to determine the edge region that should be enhanced and flat filtered.

The evolutionary time $t_{i}$ is derived by converting the scale parameter in pixels $\sigma_{i}$.

(4) $$t_{i}=\frac{1}{2\sigma_{i}^{2}},i=0\ldots M$$

A nonlinear scale-space equation could be acquired using the fast explicit diffusion (FED) algorithm [31] to solve the partial differential equation of Formula (1).

(5) $$L^{i}=(I+\tau A\left(L^{i}\right))L^{i}|i=0,1,\ldots ,n-1$$

where $A\left(L^{i}\right)$ is the conductance matrix to the image encoding and is constructed by the histogram of the Gaussian filtered scale image; I is the identity matrix; and τ is step-size, which comes from the factorization of the filter [32]. The matrix $A\left(L^{i}\right)$ remains unchanged throughout the FED cycle. When the FED loop ends, the algorithm recalculates the value of the matrix $A\left(L^{i}\right)$.

**Figure 2:**
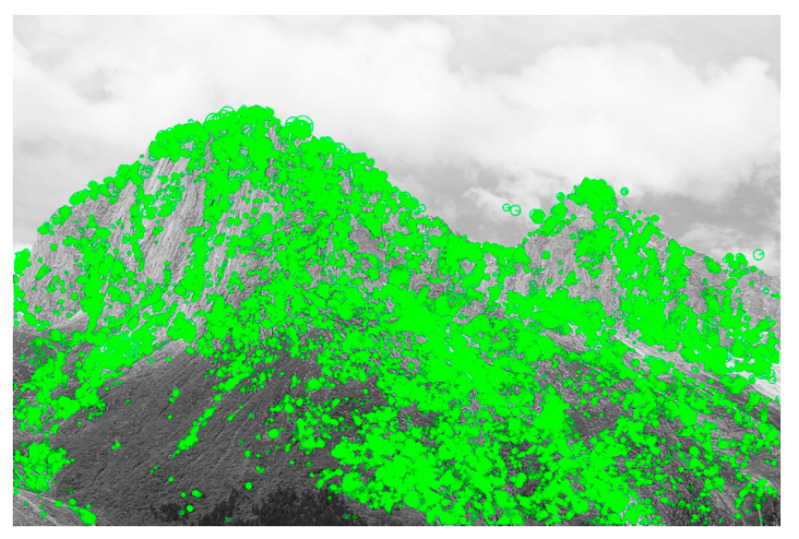
The feature extraction using the AKAZE algorithm.

After the nonlinear scale space is constructed, the Hessian matrix is used to extract the feature points. Meanwhile, SIFT is used to compare whether 26 points of the same position two layers above and below (include the current layer) are still extreme points. With this method, local feature points are extracted. To verify the feature extraction effect of the AKAZE algorithm, a slop image taken in Aba Tibetan, Sichuan, China was used to display the extraction results of feature points, shown in Figure 2.

**Feature matching.** After the feature extraction, the neighborhood matching is established to find all matching points. Euclidean distance is adopted to screen the feature point pairs. If the result does not meet the threshold, it would be removed.

In this step, the FLANN feature point matching algorithm is adopted through the K-Dimensional Tree (KD-Tree) in achieving the feature point search first, then the matching degree is determined according to the Euclidean distance formula. This method could segment the feature points of different spaces and obtained the matching point pairs in different spatial domains effectively.

Mismatched elimination. Exact matching is conducted with the Random Sampling Consistency (RANSAC) algorithm. This step allows to obtain the transformation relationship between images [33].

The idea of the RANSAC algorithm is as follows: (1) the data consist of inliers; (2) outliers are prohibited to fit the model; (3) other data are noise points. RANSAC enables to estimate high-precision parameters from a data set containing a large number of outliers, which is an excellent mismatched elimination method.

### 3.3. Sparse Reconstruction

**Figure 3:**
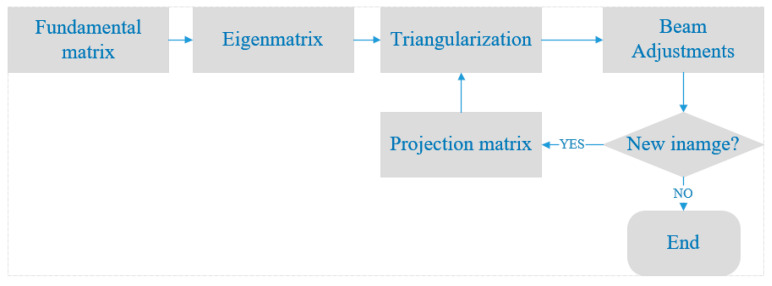
The schematic of sparse reconstruction for two images.

Figure 3 shows the schematic of sparse reconstruction for two images.

After the interior orientation parameters obtained by camera self-calibration, the exterior parameters of the structure need to be solved. Set the world coordinate system to coincide with the camera coordinate system in the first image, so that the first image could be expressed as R=I. T is the translation vector, $T={\left(0,0,0\right)}^{T}$, and R is the rotation matrix. The projection matrix $P_{1}$ of the first image is shown as Formula (6):

(6) $$P_{1}=K\left[I|0\right]=\left[K|0\right]$$

where I is the unit matrix and K is the intrinsic parameter matrix.

Similarly, the projection matrix $P_{2}$ of the second image could be represented as Formula (7):

(7) $$P_{2}=K\left[R|T\right]$$

As the eigenmatrix contains the rotation and translation matrices, it could be obtained according to Formula (8):

(8) $$E=K^{{}^{\prime }T}\mathrm{FK}$$

where F is the fundamental matrix, which could be obtained according to the matching points in the initial image pair.

The camera poses between the two cameras could be obtained by the singular value decomposition (SVD) of the eigenmatrix, Formula (9):

(9) $$E=UDV^{T}$$

Generally, U and V are orthogonal matrices of order 3, and D is the diagonal matrix.

**Figure 4:**
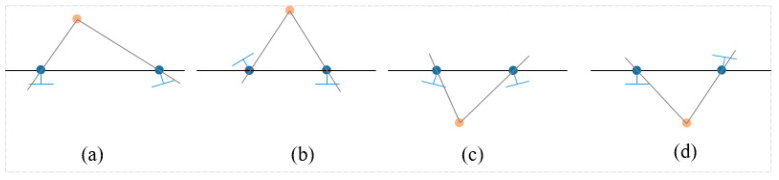
The four possible camera poses.

There are four possible solutions to the projection matrix restored by the eigenmatrix (see in Figure 4 and Formula (10)):

(10) $$P_{2}=\left[UWV^{T}|u_{3}\right];\left[UWV^{T}|-u_{3}\right];\left[UW^{T}V^{T}|u_{3}\right];\left[UW^{T}V^{T}|-u_{3}\right]$$

where $u_{3}$ is the last column of the matrix $W=\left[\begin{array}{ccc} 0 & -1 & 0\\ 1 & 0 & 0\\ 0 & 0 & 1 \end{array}\right]$.

Three-dimensional points could be calculated through the position information of the points from the projection matrices of $P_{1}$ and $P_{2}$. This process is called triangulation.

Suppose $P_{1k}$ and $P_{2k}$ are row vectors of $P_{1}$ and $P_{2}$, respectively. $M_{w}={\left(X_{w},Y_{w},Z_{w},1\right)}^{T}$ is the space coordinates of point M. ${\left(u_{1},v_{1},1\right)}^{T}$ and ${\left(u_{2},v_{2},2\right)}^{T}$ are the image coordinates of image 1 and image 2, respectively. The linear equations are obtained as Formula (11) using the coordinate system transforming relations.

(11) $$\left[\begin{array}{c} \begin{array}{c} P_{13}u_{1}-P_{11}\\ P_{13}v_{1}-P_{12}\\ P_{23}u_{2}-P_{21} \end{array}\\ P_{23}v_{2}-P_{22} \end{array}\right]M_{w}=\vec{0}$$

As the number of equations in Formula (11) is more than the unknowns, the least square is introduced to solve the space coordinates of point M. Errors may exist in the obtained coordinates as a result of the error in feature matching. Therefore, the beam adjustment (BA) is introduced to further improve the precision of coordinates because it enables to optimize the camera parameters and 3D coordinates by minimizing the error of reprojection.

The BA needs to be initialized with a good image pair. Firstly, the first BA is performed for the two initialized images. Then, add new images cyclically for a new BA. The BA is an iterative process in which all valid images are computed continuously until the end of the iteration. Finally, camera parameters and scene geometry information are obtained. Reprojection errors are the distances between projection points and real points in images. For *m* images and *n* trajectory points, the reprojection error is shown as Formula (12):

(12) $$\min\sum_{k=1}^{m}\sum_{i=1}^{n}{\left\|x_{ki}-P_{k}X_{i}\right\|}^{2}$$

where $P_{k}$ is the projection matrix and $x_{ki}$ is the image position of pint i in image k. The purpose of the BA is to minimize this function.

Multiple images reconstructing is consistent with the reconstruction of two images. After the initial projection matrix P is solved, use Formula (11) to recover the 3D coordinates of the other image matching points in the nth images. However, as the number of images increases, the difference between the newly added images and the previous images becomes larger. Moreover, the fewer the image matching pairs, the more difficult it is to calculate the fundamental matrix.

Therefore, the projection matrix P is calculated on the position of the newly added images through the reconstructed 3D point coordinates. Suppose $\left(u_{1},v_{1},1\right)$ is the image coordinates of space point $M_{i}={\left(X_{i},Y_{i},Z_{i},1\right)}^{T}$ in newly added images, the equation could be derived in Formula (13):

(13) $$\left[\begin{array}{ccc} 0^{T} & -M_{i}^{T} & v_{i}M_{i}^{T}\\ M_{i}^{T} & 0^{T} & -u_{i}M_{i}^{T} \end{array}\right]\left[\begin{array}{c} P_{i1}\\ P_{i2}\\ P_{i3} \end{array}\right]=\vec{0}$$

The projection matrix has 11 degrees of freedom (DOF). The projection matrix of the nth images could be obtained using the projections of six reconstructed 3D points on the new image. When more than six points satisfy this requirement, RANSAC could be helpful for a more accurate projection matrix.

### 3.4. Dense Reconstruction

The sparse point cloud is only useful for regular objects with obvious features, but fails to present the surface information of the object well. Therefore, the complex scenarios need a denser point cloud, for example, rock engineering. The patched-based multi-view stereo (PMVS) algorithm [34] could reconstruct high-precision models with rich surface details for scenes with unclear texture, limited point view, large curvature, and so on.

Patch is a rectangle of a local tangent plane that approximates the object’s surface. V(p) is defined as the image set of containing all visible patches. R(p) is the patches set of reference images, R(p)∈V(p). The discrepancy function could be defined as Formula (14):

(14) $$g\left(p\right)=\frac{1}{\left|V\left(p\right)-R\left(p\right)\right|}\underset{I\in \left(V\left(p\right)-R\left(p\right)\right)}{\sum }h\left(p,I,R\left(p\right)\right)$$

where, V(p)−R(p) means patches of V(p) that remove R(p), and $h\left(p,I_{1},I_{2}\right)$ is the grayscale consistency function of I and R(p). The steps of the solution are as follows [35,36]:

(1) Divide patch p into smaller squares, u×u.
(2) Calculate the difference value of patch p on the image $I_{i}$ to obtain the pixel gray $q(p,I_{i})$, through bilinear interpolation.
(3) Subtract the normalized cross correlation (NCC) value of q(p,$I_{1}$) and q(p,$I_{1}$) from 1.
(4) Initialize and optimize the relevant parameters.

The continuity of the patches is a major disadvantage. To solve this problem, the image $I_{i}$ is divided into many $\beta_{1}\times \beta_{1}$ pixel pieces $C_{i}\left(x,y\right)$, where i is the ith image and (x,y) is the subscript of an image piece. For a patch p and the corresponding set V(p), project p onto the image of V(p) to obtain the image piece corresponding to patch p. Set $Q_{i}\left(x,y\right)$ records all the patches projected onto the image pieces.

## 4. Experiment Preparation

A series of experiments for 3D reconstruction based on images from different angles and directions using the A-SfM algorithm were performed.

Three experiments were designed. Experiment 1 was used to evaluate the results of 3D reconstruction with proposed A-SfM. The second one was employed to test the accuracy of A-SfM. Experiment 3 was conducted to detect the deformation of rock mass surface.

**Figure 5:**
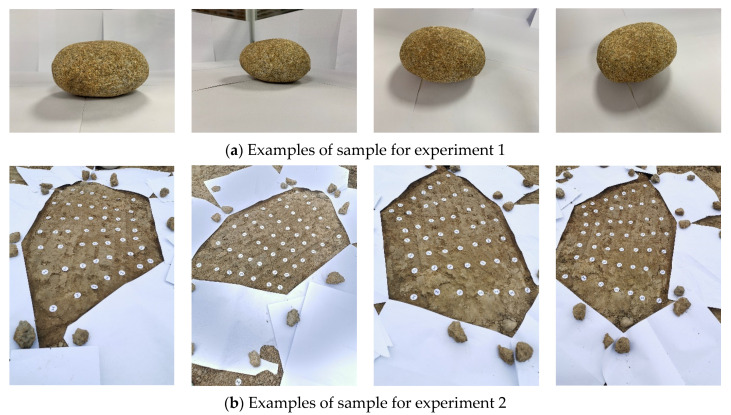
Examples of image samples.

**Experiment** **1.** *Two groups of images were acquired: (1) the rock in the indoor environment without interference; (2) the surface of the soil outdoors. The number of the images in the two groups was 32 and 16, respectively. The iPhone XR camera is selected to acquire images in a counterclockwise direction around the objects, with the distance between the object and camera of 2 m. The examples of image samples are shown in Figure 5.*

**Figure 6:**
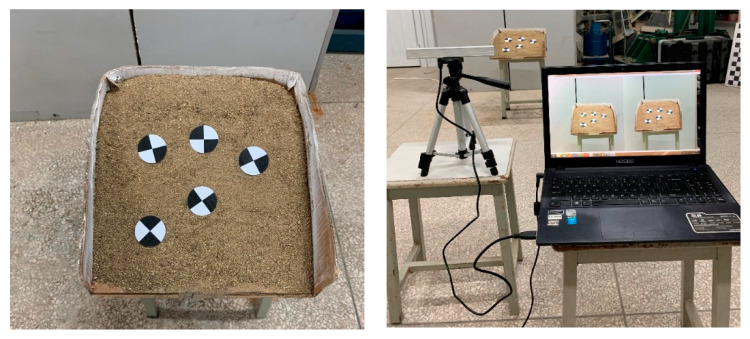
The slope model built in a laboratory environment.

**Experiment** **2.** *A slope model was built in a laboratory environment to explore the accuracy of the A-SfM algorithm, as shown in Figure 6. The dimension of the model is 35 cm in length, 35.5 cm in width, 12 cm in height, and 50 in gradient. The component mainly consists of the sand, low-grade gravel, and a small amount of mudstone. Mark points were used for binocular vision monitoring to serve as reference data for A-SfM analysis. Three groups of tests were designed to distinguish different mark points and different photograph distances:*

*Group 1: The photograph distance was 2 m, and the mark points were common.*

*Group 2: The photograph distance was 1 m, and the mark points were common.*

*Group 3: The photograph distance was 1 m, and the mark points were concentric circles.*

**Figure 7:**
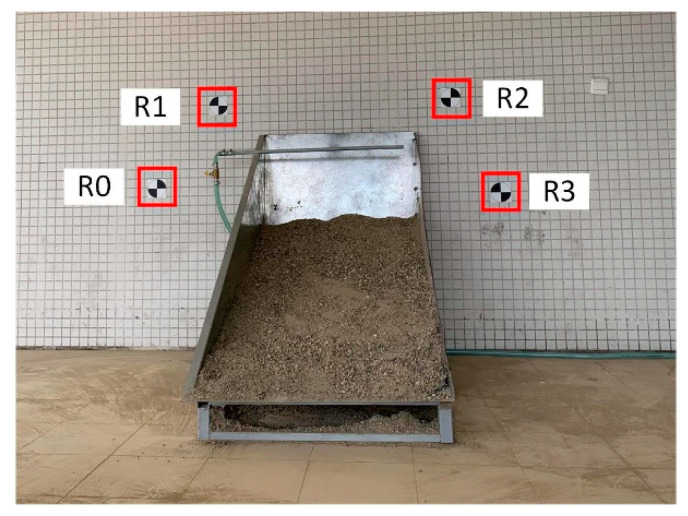
Layout of geodetic control points.

**Experiment** **3.** *The surface deformation of rock mass was quantified based on the 3D reconstruction results of the surface before and after the disturbance. Geodetic control points were applied to compare the two results in the same coordinate system (Figure 7). The procedure was as follows:*

*(1)*  *The geodetic control points were measured and recorded with the total station electronic tachometer.*
*(2)*  *The distance between the wall and each image capture station was measured using a laser range finder, and the locations were marked.*
*(3)*  *Eight images before the disturbance were captured sequentially.*
*(4)*  *Four sandstone samples of 50 mm in diameter and 50 mm in height were used to simulate the uplift and deformation. The samples were placed lightly on the model to avoid disturbing the rock mass at other locations.*
*(5)*  *Eight images after the disturbance were captured sequentially.*

## 5. Results and Analysis

### 5.1. Description of Results

#### 5.1.1. Results for Experiment 1

**Figure 8:**
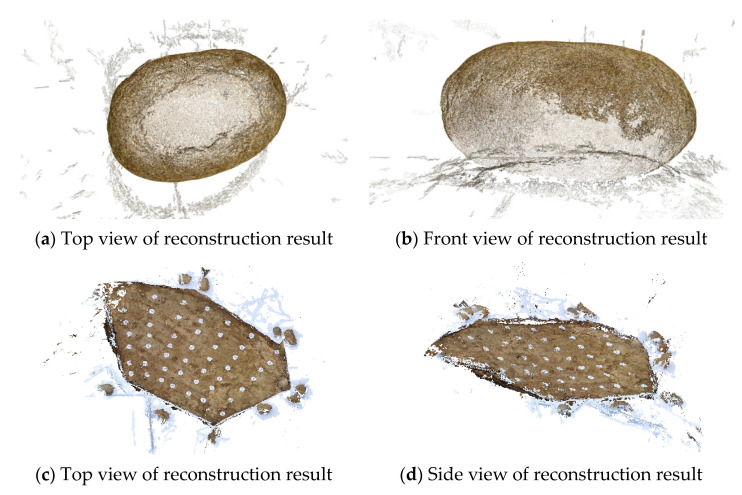
The 3D reconstruction results using the A-SfM (structure from motion) algorithm in Experiment 1.

Figure 8 shows the 3D reconstruction results with the proposed A-SfM algorithm, where (a) and (b) displays the reconstruction results of the first group images, and (c) and (d) are the results of the second group.

#### 5.1.2. Results for Experiment 2

The results of binocular vision measurement were served as reference data for accurate analysis of the reconstructions. Studies related to binocular vision measurements have been completed and published [5,37]. The measurement results, as shown in Table 4, Table 5 and Table 6, were obtained through camera calibration, pixel coordinate positioning, and space coordinate calculation.

**Figure 9:**
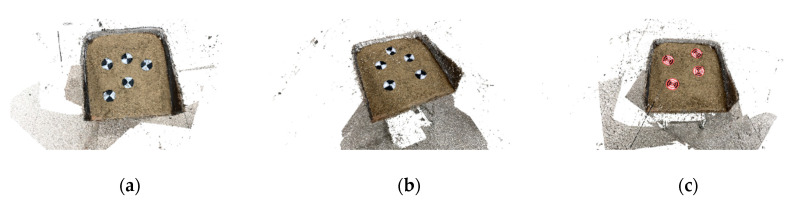
Results of A-SfM reconstruction in Experiment 2: (**a**) the photograph distance was 2 m, and the mark points were common; (**b**) the photograph distance was 1 m, and the mark points were common; (**c**) the photograph distance was 1 m, and the mark points were concentric.

Two methods, SfM and A-SfM, were used to establish the 3-D reconstruction, and the results of A-SfM are shown in Figure 9.

#### 5.1.3. Results for Experiment 3

**Figure 10:**
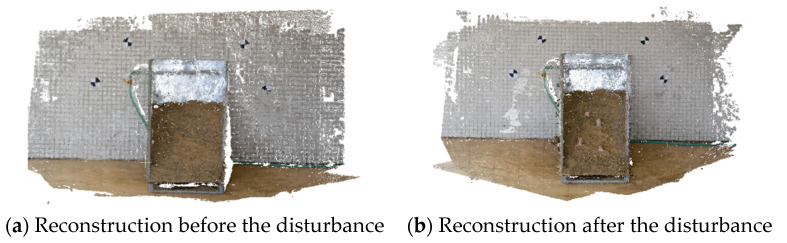
Reconstruction before and after the disturbance.

The aforementioned images captured before and after the disturbance were then computerized and reconstructions using A-SfM before and after the disturbance were established, as shown in Figure 10.

### 5.2. Reconstruction Results Analysis

The results of Experiment 1 showed that two groups of images were reconstructed well, and the reconstructed models were almost identical to the real objects. However, there were still some disturbance point clouds that affect the modeling. The disturbance point clouds were predominated by the environmental features of shadows and interferers, which were apparent in multiple images. This affected the eigenmatrix of the object and reconstruction of the contour. Besides, the first image took about 80 times longer than others in feature extraction because it needed more time in selecting the initial relative features. It could be inferred that the results of reconstruction using the A-SfM performed well in an environment with prominent characteristics and strong contrast, for example, the rock engineer environment studied in this paper. This gives rise to the application value of rock mass surface detection. Even so, the model accuracy needed further discussion, which is why Experiment 2 was designed.

In Experiment 2, to evaluate the accuracy of the A-SfM algorithm, the results of reconstructions should be compared with those of binocular vision measurements. However, the distance calculated by the reconstructing results is the relative distance instead of the actual measured distance in the space coordinate system. The scaling factor is introduced to map the relative distances into physical distances with Formula (15) [38]:

(15) $$S=\frac{d_{known}}{I_{known}}$$

where S is the scaling factor, $d_{known}$ is the physical length of an object, and $I_{known}$ is the pixel length of the object on the imaging plane. Table 7 lists the calculated scaling factor.

According to the scaling factor and the pixel length, the physical length between the mark points in the two reconstructions, one for SfM and the other for A-SfM, was calculated, as shown in Table 8 and Table 9.

**Figure 11:**
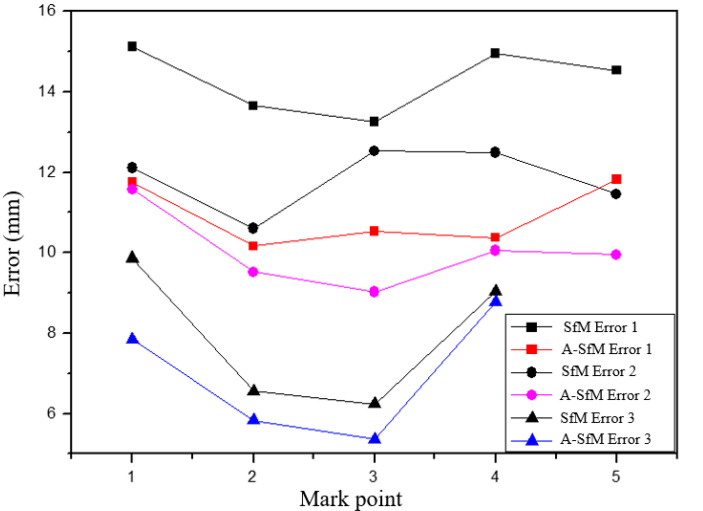
The reconstruction errors of the three groups of tests.

Table 10 shows the results of comparing the two reconstructions, using SfM and A-SfM, with the binocular vision measurements. Comparing with the results of reconstructions (both SfM and A-SfM) with those of binocular vision measurement, the 3D reconstruction performance of SfM before and after the improvement was verified. Moreover, the improved SfM algorithm significantly promoted the measurement accuracy, which effectively reflects the real situation (Figure 11). The measurement accuracy was improved from 2.7 mm to 4.58 mm in group 1; from 0.53 mm to 3.51 mm in group 2; and from 0.25 mm to 2.01 mm in group 3.

### 5.3. Rock Mass Surface Deformation Analysis

Different from most of the traditional measuring methods of single point deformation detection, the 3D point cloud could qualify the variation of the whole monitoring region. The surface deformation of rock mass could be quantified with the deformation detection by the 3D reconstruction results of the surface before and after the disturbance. This proposed procedure included point cloud data cleaning, geodetic control point registration, iterative closest point (ICP) registration, Euclidean distance calculation between registration, and reference point clouds.

**Figure 12:**
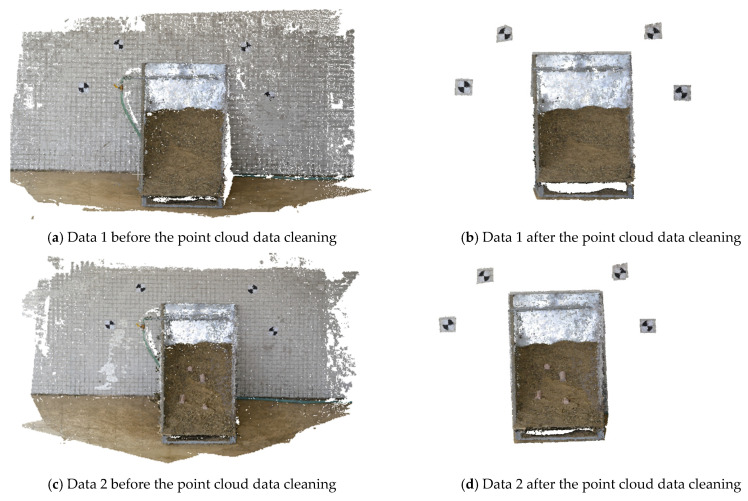
Results before and after the point cloud data cleaning.

**Point cloud data cleaning.** As discussed in Experiment 1, numerous invalid points and outliers would be generated when 3D point cloud data reconstructed by A-SfM were imported into CloudCompare. Because only the detected deformation area should be preserved, the point cloud data could first be preprocessed through the Bounding Box algorithm. Then, outlier data could be eliminated using the statistical analysis filter. Finally, the invalid points could be cut and divided manually. Figure 12 shows the results before and after the point cloud data cleaning, shown here for the reconstruction before the disturbed data 1 and after the disturbed data 2.

**Figure 13:**
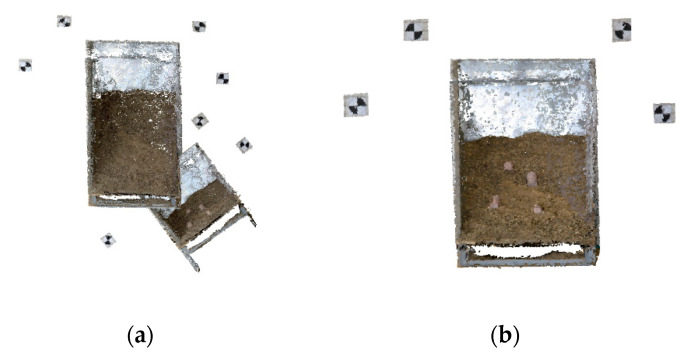
Results before and after the geodetic control point registration. (**a**) The position and distribution of data before the geodetic control point registration, and (**b**) The data after the geodetic control point registration.

**Geodetic control point registration.** To make the 3D point clouds before and after the disturbance in the same world coordinates system, the geodetic control point in the reconstructions was registered after the point cloud data cleaning. This proposed procedure took the cloud data before the disturbance as the reference point and the cloud data after the disturbance as the matching point. Figure 13 shows the results of geodetic control point registration, where (a) represents the position and distribution of data before the geodetic control point registration, and (b) is the data after the geodetic control point registration.

**Figure 14:**
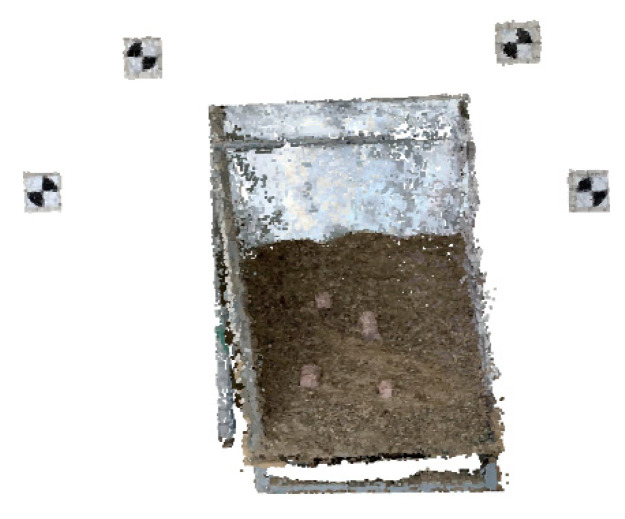
The results after iterative closest point (ICP) registration.

**Iterative closest point (ICP) registration.** There were some errors because the control points were manually selected during the geodetic control point registration. Therefore, it was necessary to use ICP for precise registration. The cloud data before the disturbance were defined as the reference point, and the cloud data after the disturbance were defined as the matching point. Figure 14 presents the results after ICP registration.

**Figure 15:**
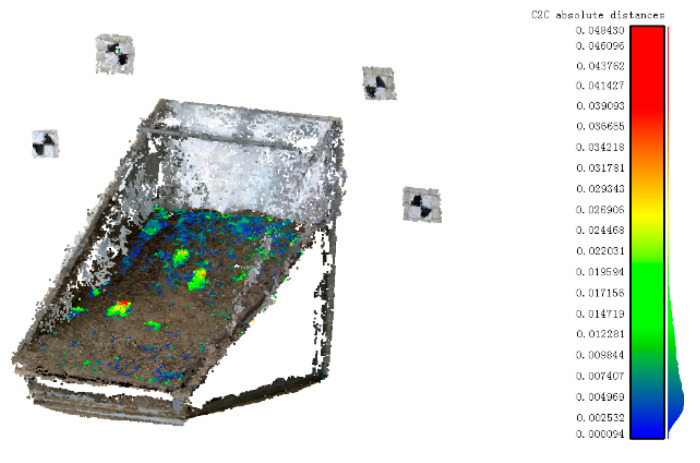
Rock mass surface deformation detection.

**Rock mass surface deformation detection.** The Euclidean distance between points and neighbor points was calculated using the precise registration data. The deformation ranged from 8.97 × 10^−5^ m to 0.61 × 10^−1^ m, and the color scale is shown in Figure 15.

**Results analysis.** The uplift to simulate the deformation was rock samples with a diameter of 50 mm. However, the lower part of the rock was slightly inserted into the rock mass model and the upper part was placed on the surface of the model. It can be seen in Figure 15 that the smallest surface deformation in the undisturbed zones is 0.094 mm, whereas the maximum deformation in disturbed zones is 48.43 mm. The results presented were generally with the actual situation.

## 6. Conclusions

Three-dimensional reconstruction of rock mass surface is a crucial step in surface deformation detection, which could assist in understanding rock mass progressive failure processes. On the basis of the SfM method, an A-SfM method was proposed for rock engineering applications so as to acquire the 3D reconstruction that is suitable for the characteristics of rock mass surface. The AKAZE algorithm is used to improve the structure flow of SfM so as to extract the features of the rock mass more easily at close range. Three experiments verified the ability of the proposed A-SfM method. The specific conclusions can be drawn as follows:

(1) The results of 3D reconstruction in Experiment 1 using the proposed A-SfM showed the reconstructed models were almost identical to the real objects.
(2) In Experiment 2, the measurement accuracy of the A-SfM improves compared with the measurement accuracy of the SfM.
(3) Experiment 3 shows that the results detected were generally consistent with the actual situation. The deformation detection by the 3D reconstruction results of the surface before and after the disturbance confirmed that the proposed method effectively quantified the surface deformation of the rock mass.

## Figures and Tables

**Table 1:** Average time cost and average calculation cost of five types of operators.

Algorithm Type	SIFT	SURF	ORB	AKAZE	BRISK
Average time cost (ms)	6877.32	1205.54	571.90	9189.28	199,348.45
Average calculated cost (%)	75.20	74.48	65.78	66.49	77.01

**Table 2:** Average number of effective feature points and accuracy of five types of operators.

Algorithm Type	Number of Feature Points	Number of Interior Points	Number of Matching Points	Efficiency of Feature Points (%)	Matching Accuracy (%)
SIFT	107,326	44,211	48,756	45.43	90.68
SURF	30,259	9370	11,032	36.46	84.93
ORB	15,075	4224	5022	33.31	84.10
AKAZE	31,685	14,553	15,971	50.40	91.12
BRISK	131,602	55,573	61,584	46.80	90.24

**Table 3:** The related technical specifications of sensors for image acquisition.

Sensors	Effective Pixels (MP)	Resolution (Pixels)	Focal Length (mm)	Types of Sensors	Weight
iPhone 6 Plus	7.99	2449 × 3264	29	Complementary Metal Oxide Semiconductor (CMOS)	172 g
iPhone 6s	-	2449 × 3265	29	CMOS	143 g
iPhone XR	-	4032 × 3024	26		194 g
Panasonic Lumix LX5	9.52	2520 × 3776	24~90	charge coupled device (CCD)	271 g
Panasonic Lumix ZX20	14.1	3240 × 4230	24~480	CMOS	204 g
Camon EOS 7D	17.92	2345 × 5184	29~216	CMOS	820 g
Acom Trailcam 5310	5.0~12.0	4000 × 3000	6	CMOS	245 g
Nikon 750	24.3	6016 × 4016	24~120	CMOS	750 g
Nikon D3100	14.2	4608 × 3072	18~200	CMOS	455 g

**Table 4:** Binocular vision measurements from group 1.

Frames	Image Coordinates for the Left Camera		Image Coordinates for the Right Camera		Space Coordinates			Physical Length (mm)
	*u*	*v*	*u*	*v*	*x*	*y*	*z*	
1	319.264	194.168	253.782	183.666	72.4124	−28.508	2129.84	92.69611
2	389.689	194.786	323.589	181.813	165.042	−28.4272	2126.33	101.072
3	455.172	208.376	387.218	194.786	244.194	−9.80191	2066.3	98.59255
4	387.836	231.234	319.882	217.643	155.528	18.482	2033.76	99.2226
5	319.882	242.353	251.311	230.616	68.3561	32.1841	1988.39	153.9743

**Table 5:** Binocular vision measurements from group 2.

Frames	Image Coordinates for the Left Camera		Image Coordinates for the Right Camera		Space Coordinates			Physical Length (mm)
	*u*	*v*	*u*	*v*	*x*	*y*	*z*	
1	382.276	223.82	296.407	213.936	36.4941	39.9201	1470.81	117.3779
2	304.438	259.651	215.481	249.149	33.6499	88.4536	1377.29	105.402
3	303.82	320.191	210.539	307.836	178.935	87.0295	1392.37	146.0726
4	471.851	317.102	377.952	302.276	175.729	41.2305	1482.06	100.7577
5	455.79	260.268	366.214	247.913	113.495	8.54642	1553.66	100.3388

**Table 6:** Binocular vision measurements from group 3.

Frames	Image Coordinates for the Left Camera		Image Coordinates for the Right Camera		Space Coordinates			Physical Legth (mm)
	*u*	*v*	*u*	*v*	*x*	*y*	*z*	
1	272.315	270.77	174.708	260.886	6.48721	44.9195	1320.11	160.9808
2	455.172	245.442	358.183	231.851	160.852	24.7255	1361.08	110.4423
3	458.261	311.542	355.712	297.952	151.693	74.9193	1263.13	152.3925
4	285.905	356.639	180.886	344.902	113.495	8.54642	1553.66	134.0647

**Table 7:** The scaling factor for each group. Sfm, structure from motion.

Groups	Physical Length (mm)	Pixel Length (pixels)		Scaling Factor	
		SFM	A-SFM	SFM	A-SFM
Experiment 1	40	0.203489	0.202819	196.5708	197.2201
Experiment 2	40	0.16536	0.165183	241.8965	242.1563
Experiment 3	50	1.04286	1.039834	47.94507	48.0846

**Table 8:** Pixel length and physical length between the mark points in reconstruction using SfM.

No.	Group 1		Group 2		Group 3	
	Pixel Length (pixels)	Physical Length (mm)	Pixel Length (pixels)	Physical Length (mm)	Pixel Length (pixels)	Physical Length (mm)
1	0.394639	77.5745126	0.435196	105.2724	3.15192	151.119
2	0.444712	87.4174034	0.391893	94.79753	2.1669	103.8922
3	0.43411	85.3333595	0.552053	133.5397	3.04844	146.1577
4	0.428673	84.264604	0.36491	88.27044	2.60767	125.0249
5	0.709373	139.442034	0.367464	88.88824	-	-

**Table 9:** Pixel length and physical length between the mark points in reconstruction using A-SfM.

No.	Group 1		Group 2		Group 3	
	Pixel Length (pixels)	Physical Length (mm)	Pixel Length (pixels)	Pixel Length (pixels)	Physical Length (mm)	Pixel Length (pixels)
1	0.410428	80.94468	0.43693	105.8052	3.18461	153.1307
2	0.460962	90.91099	0.39595	95.8819	2.175576	104.6117
3	0.446506	88.05996	0.565952	137.0488	3.057618	147.0243
4	0.450515	88.85052	0.374568	90.70391	2.605403	125.2798
5	0.720824	142.161	0.373274	90.39073	-	-

**Table 10:** Results of comparing the two reconstructions with the binocular vision measurements.

Groups	Binocular Vision	SFM	A-SFM	Error 1	Error 2	Accuracy Improvement
Experiment 1	92.69611	77.57451	80.94468	15.1216	11.75143	3.370167
	101.072	87.4174	90.91099	13.65456	10.16098	3.493582
	98.59255	85.33336	88.05996	13.25919	10.53259	2.726596
	99.2226	84.2646	88.85052	14.958	10.37208	4.585919
	153.9743	139.442	142.161	14.53227	11.81327	2.719006
Experiment 2	117.3779	105.2724	105.8052	12.10551	11.57264	0.532871
	105.402	94.79753	95.8819	10.60446	9.520093	1.084369
	146.0726	133.5397	137.0488	12.5329	9.023786	3.50911
	100.7577	88.27044	90.70391	12.4873	10.05383	2.433472
	100.3388	88.88824	90.39073	11.45058	9.94809	1.502491
Experiment 3	160.9808	151.119	153.1307	9.86179	7.85013	2.01166
	110.4423	103.8922	104.6117	6.550145	5.830614	0.719531
	152.3925	146.1577	147.0243	6.234797	5.368136	0.866661
	134.0647	125.0249	125.2798	9.039812	8.784983	0.254829
